# Development and validation of an infrared-artificial intelligence software for breast cancer detection

**DOI:** 10.37349/etat.2023.00135

**Published:** 2023-04-27

**Authors:** Enrique Martín-Del-Campo-Mena, Pedro A. Sánchez-Méndez, Eva Ruvalcaba-Limon, Federico M. Lazcano-Ramírez, Andrés Hernández-Santiago, Jorge A. Juárez-Aburto, Kictzia Y. Larios-Cruz, L. Enrique Hernández-Gómez, J. Andrei Merino-González, Yessica González-Mejía

**Affiliations:** 1 Oncologic surgery, State Cancer Center: Miguel Dorantes Mesa, Aguascalientes 100, Progreso Macuiltepetl, Xalapa, Veracruz 91130, Mexico; 2 Hearthcore SAPI de CV, Bosques de Tabasco 79, Bosques de México, Tlalnepantla, Mexico State 91130, Mexico; 3 Teaching and Research, Breast Cancer Foundation (FUCAM, A.C.), Av. Bordo 100, Viejo Ejido de Santa Úrsula Coapa, Coyoacán, Mexico City 04980, Mexico; 4 Epidemiological Surveillance and Preventive Medicine, General Hospital Dr. Fernando Quiroz Gutiérrez, ISSSTE, Felipe Ángeles y Canario s/n, Bellavista, Álvaro Obregón, Mexico City 01140, Mexico; 5 Radiology, Breast Cancer Foundation (FUCAM, A.C.), Av. Bordo 100, Viejo Ejido de Santa Úrsula Coapa, Coyoacán, Mexico City 04980, Mexico; University of Campania L. “Vanvitelli”, Italy

**Keywords:** Breast cancer, infrared thermography, artificial intelligence, mammography, screening

## Abstract

**Aim:**

In countries where access to mammography equipment and skilled personnel is limited, most breast cancer (BC) cases are detected in locally advanced stages. Infrared breast thermography is recognized as an adjunctive technique for the detection of BC due to its advantages such as safety (by not emitting ionizing radiation nor applying any stress to the breast), portability, and low cost. Improved by advanced computational analytics techniques, infrared thermography could be a valuable complementary screening technique to detect BC at early stages. In this work, an infrared-artificial intelligence (AI) software was developed and evaluated to help physicians to identify potential BC cases.

**Methods:**

Several AI algorithms were developed and evaluated, which were learned from a proprietary database of 2,700 patients, with BC cases that were confirmed through mammography, ultrasound, and biopsy. Following by evaluation of the algorithms, the best AI algorithm (infrared-AI software) was submitted to a clinic validation process in which its ability to detect BC was compared to mammography evaluations in a double-blind test.

**Results:**

The infrared-AI software demonstrated efficiency values of 94.87% sensitivity, 72.26% specificity, 30.08% positive predictive value (PPV), and 99.12% negative predictive value (NPV), whereas the reference mammography evaluation reached 100% sensitivity, 97.10% specificity, 81.25% PPV, and 100% NPV.

**Conclusions:**

The infrared-AI software here developed shows high BC sensitivity (94.87%) and high NPV (99.12%). Therefore, it is proposed as a complementary screening tool for BC.

## Introduction

In Mexico, over 75% of new breast cancer (BC) cases are detected in locally advanced stages, which increases treatment costs and reduces overall survival [1]. Two of the main reasons for low rates of early BC detection are the lack of mammography units and few specialized personnel that can interpret mammograms.

Mexico is a member country of the Organization for the Economic Co-operation and Development (OECD) with the least amount of mammography units per million inhabitants (9.7), meanwhile, countries like South Korea and the United States have 61.6 and 65.3, respectively [2]. In 2015, the Mexican Council of Radiology and Image reported 3,911 certified medical radiologists in Mexico [3]. Similarly, in 2018, the Mexican Medical Journal reported 3,819 physicians with a specialization in diagnostic-therapeutic medical imaging [4] 3.06 per 100,000 habitants, which is particularly low compared to the European average (12.8) [5] and the US (8.43) [6]. It is therefore crucial to strengthen the investment and development of new techniques that allow for more patients to be screened and facilitate earlier detection of BC at lower costs, particularly for rural and isolated regions.

Infrared thermography is a complementary technique for BC screening. Approved by Food and Drug Administration (FDA) [7], this technique has been used extensively in research to detect BC [8–10]. The advantages of using infrared thermography for BC screening include its safety since it does not emit ionizing radiation or submit the breast to mechanical pressure, as well as low cost and portability.

The use of infrared thermography is based on the principle that malignant tumors stimulate the formation of new blood vessels to nourish and sustain accelerated cellular growth, which is called angiogenesis [11], producing an increase in blood flow and therefore in the temperature around the tumor. This temperature change produces a thermal asymmetry between both breasts, which is shown as a higher surface temperature in the breast that has the tumor. The temperature difference between breasts may vary and is different for each patient, although some studies mention that the common temperature differences for BC are above 2°C [12].

The objective of this research was to develop artificial intelligence (AI) software to detect BC from infrared images (infrared-AI software), learned from known BC cases, as detected by mammography, ultrasound, and biopsy. The infrared-AI software was then evaluated as a BC screening test, in comparison to mammography evaluations.

## Materials and methods

### Patients

A total of 3,812 female participants were recruited for this research (≥ 18 years old). All participants attended the Breast Cancer Foundation (FUCAM A.C.) in Mexico City between June 2018 and April 2021 to receive screening mammography or complementary tests due to suspicion of BC.

Exclusion criteria in the trials included pregnancy, previous BC diagnosis, currently undergoing BC treatment, breast implants, partial or total mastectomy, breast surgery or surgery in the thorax area within the previous two years, and/or a breast biopsy in the previous three months. During the test, patients were excluded if: they did not receive mammography after the initial infrared test; they only received a breast ultrasound, tests reported them as breast imaging reporting and data system (BI-RADS) 3,4 or 5 without follow-up at FUCAM A.C. to confirm BC; they did not receive radiologic or histopathologic evaluations, or infrared tests were poorly taken.

This research was approved by the Research, Bioethics, and Biosafety Committee from FUCAM A.C. All participants signed informed consent.

### Infrared tests

**Figure 1.**
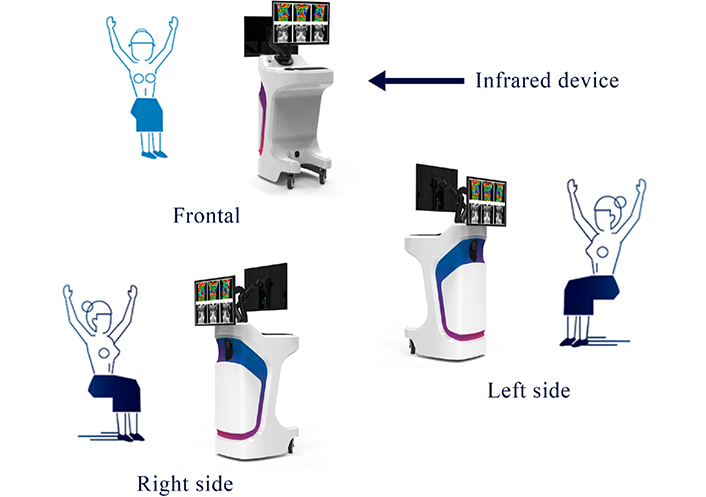
Infrared test image acquisition

**Figure 2.**
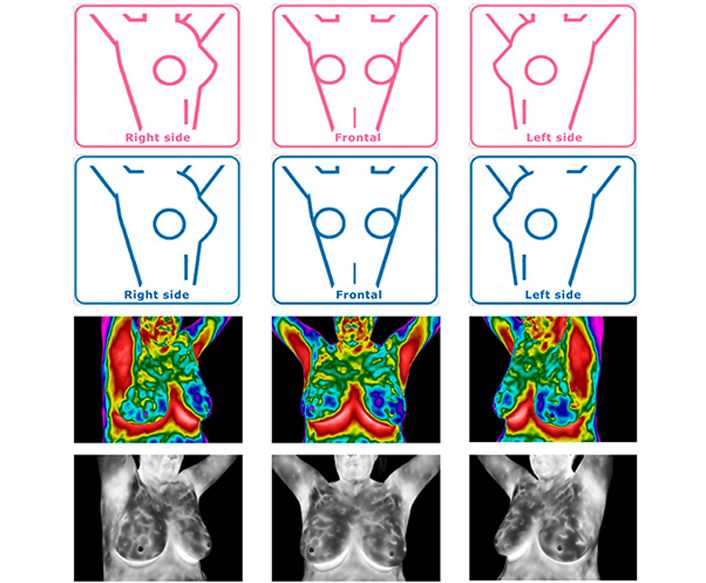
Infrared test reference positions and example images

Prior to the infrared test, each patient answered a questionnaire about their family history, symptoms, previous activities, and conditions that affect their body temperatures, such as drug and/or stimulant intake, and current menstrual cycle phase (Supplementary material). The infrared tests were performed before the mammography, ultrasound, and/or biopsy, following the guidance of the American Academy of Thermology [13], with the allowed adjustments (Figure 1). Three basal images were taken in the first image set: frontal, 30° to the right of midline, and 30° to the left. The second image set called the dynamic test, was performed to identify which areas retained a higher temperature after cooling the breast, armpit, and neck areas using disposable hypoallergenic wet tissues. Following 1 min of rest after cooling to allow the remaining liquid to evaporate, the second set of images was taken from the same positions as the first image set (Figure 2). For patients with recent mammography or ultrasound, the infrared test was performed between 24 h and 30 days after those tests.

The six infrared images and the clinical data made up a registry for each patient. Registries were stored on a software database made for this purpose. Infrared tests were performed by four nurses trained to use the infrared camera and software. The infrared camera used was a FLIR^®^ T650sc, with an infrared resolution of 640 pixels × 480 pixels, thermal sensitivity of < 20 mK (0.02°C), and accuracy of ± 1°C.

### Research study’s design

The research study was divided into two phases: (1) the development of the AI algorithms, and (2) clinical validation. In the second phase, a double-blind design was implemented, where the infrared test operator did not know the mammography’s results, and the mammography technicians and radiologists did not know the infrared test’s results.

### Phase 1: development of the AI algorithm

To find the best AI model for these trials, several AI algorithms were developed and tested. The algorithms developed included architectures based on neural networks (multi-layered, convolutional, and deep learning), support vector machines (SVMs), and K-nearest neighbors. These AI algorithms were supplied with a subset of patient registries as well as the mammograms’ BI-RADS score (mammograph evaluations performed by certified radiologists from FUCAM A.C.). The number of patient registries needed to obtain an efficient AI algorithm that could distinguish between patients with or without malignancy was defined by theoretical saturation. The AI algorithms were designed to generate a binary classification: zero (0) for test results that were “non-suspicious” of BC and one (1) for test results “suspicious” of BC. The 6 categories of the BI-RADS [14] scale which was used to define the BC probability of each patient are shown in Table 1.

**Table 1.** Categories of BI-RADS scales and management recommendations

**Assessment**	**Management**	**Probability of malignancy**
BI-RADS 0: not conclusive	Additional tests are requiring	N/A
BI-RADS 1: normal breast	Routine mammography screening	Essentially 0%
BI-RADS 2: benign	Routine mammography screening	Essentially 0%
BI-RADS 3: probably benign	Requires semestral follow-up by two years	≤ 2%
BI-RADS 4: probably malignant	Biopsy required	> 2% and < 95%
BI-RADS 4A: low suspicion	Biopsy required	> 2% and ≤ 10%
BI-RADS 4B: moderate suspicion	Biopsy required	> 10% and ≤ 50%
BI-RADS 4C: high suspicion	Biopsy required	> 50% and ≤ 95%
BI-RADS 5: highly suggestive of malignancy	Biopsy required	≥ 95%
BI-RADS 6: histologically confirmed malignancy	Surgical excision when clinically appropriate	N/A

N/A: not applicable

The subset of patient registries supplied to each AI algorithm was labeled as “without BC” if they belonged to patients with mammogram BI-RADS1,2, and “with BC” if they belonged to patients with mammogram BI-RADS 3,4, or 5 where BC was confirmed by biopsy. In order to obtain an AI algorithm with high BC sensitivity, the registries from patients with BI-RADS 3,4, or 5 that had a follow-up biopsy result of benign pathology, and BI-RADS 3 cases that were re-evaluated afterward as BI-RADS 2 (mammography false positives), were not supplied to the AI algorithms.

To evaluate and compare the efficiency of each AI algorithm and identify the best model, a subset of patient registries was kept aside from the development process described above. This “validation subset” was used to calculate the effectiveness metrics: sensitivity, specificity, positive predictive value (PPV) and negative predictive value (NPV), area under the curve (AUC), and F_1_ score for each algorithm. For this research, only the algorithms that showed a sensitivity equal to or superior to 95% with an F_1_ score superior to 0.75 were kept.

### Phase 2: clinical validation

In the second phase, a new group of participants underwent infrared-AI and mammography screening at FUCAM A.C. between September 2020 and April 2021. The findings from the infrared-AI software were consigned into individual reports according to the classification of the American Academy of Thermology based on the scale proposed by Villa Marie and Marseille [15]; when the AI algorithm outputs a “non-suspicious” (0) test result, it was reported as a Th1 or Th2. When the AI algorithm outputs a test result “suspicious” of BC (1), it was reported as a Th3, Th4, or Th5.

The sample size for this phase was 337 patients; this was calculated using the equation on studies for diagnostic tests proposed by Pedraza and Raad [16], with α at 5% and β at 10%. The sensitivity for the mammography applied in the development phase is π_1_ (sensitivity from the mammography applied in the development phase), and π_2_ (sensitivity from the AI algorithm validation subset) is the sensitivity from the AI algorithm validation subset.

### Statistical analysis

Descriptive statistics analysis was applied to clinical variables obtained from clinical files, radiological and histopathological reports, as well as patient questionnaires completed prior to the infrared tests. Chi [2] and Fishers’ exact tests were used to analyze the correlation between the infrared test results and the clinical variables. The statistical analyses were carried out using the software EPI Info™ 6.0 version [Centers for Disease Control and Prevention (CDC), Atlanta, USA] and web statistical calculator SISA (Daan Uitenbroek, Hilversum, Netherlands) [17].

For validation of the infrared-AI software, the algorithm’s effectiveness metrics were calculated and compared with the same scores calculated from the mammography evaluations and biopsy. A two-tailed *P* ≤ 0.05 was considered statistically significant.

## Results

**Figure 3.**
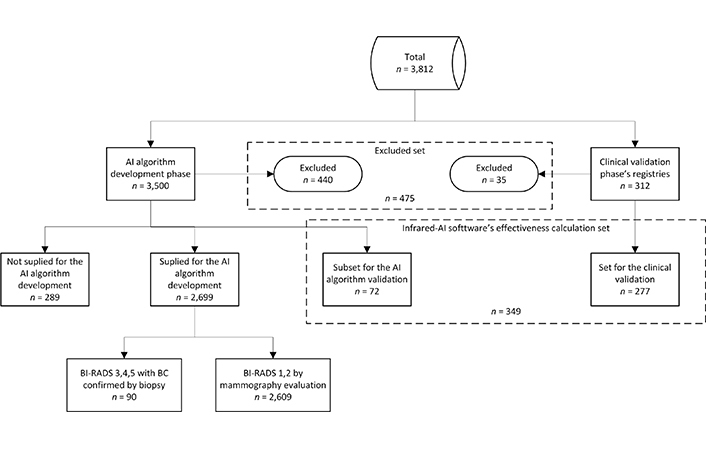
Distribution of patient registries used during the study

Registries from 3,812 patient candidates were obtained during the study (3,500 for the AI algorithm development phase, and 312 for the clinical validation phase). Of these, 440 registries were excluded during phase 1, and 35 were excluded from phase 2 due to meeting one or more of the exclusion criteria. A total sample of 3,337 patient registries (3,060 for phase 1, and 277 for phase 2) was used in this research (Figure 3).

The average patient age was 54 years, ranging from 18 years to 90 years. One in five patients reported a family history of BC and 65.1% reported at least one activity or condition that altered their body temperature. Biopsies were performed for 300 of the 3,337 participants, of which 129 (43%) were diagnosed with BC and 171 (57%) with benign breast pathology. The sociodemographic and pathological characteristics of participants are shown in Table 2.

**Table 2.** Patient sociodemographic and disease characteristics

**Variable**	**Total**
Patients	3,337
Age (years)	54 ± 10
BMI (kg/m^2^)	28.15 ± 4.73
Underweight	13 (0.4%)
Normal	896 (26.9%)
Overweight	1,396 (41.8%)
Obesity	1,032 (30.9%)
Premenopausal	1,038 (31.1%)
Postmenopausal	2,299 (68.9%)
Age of menopause (years), *n* = 2,999	47 ± 5
Family history BC/OC	
No	2,655 (79.6%)
Yes	682 (20.4%)
Breast pain	
No	2,435 (73%)
Yes	902 (27%)
Activities and conditions that affect body temperature^*^	
No	1,163 (34.9%)
Yes	2,174 (65.1%)
Breast density	
A	194 (5.8%)
B	2,584 (77.4%)
C	476 (14.3%)
D	12 (0.4%)
N/A	76 (2.1%)
Histopathological test, *n* = 300	
Benign	171 (57%)
Malignant	129 (43%)
BC grade, *n* = 129	
I	9 (7%)
II	69 (53.5%)
III	36 (27.9%)
N/A	15 (11.6%)
Tumor size^#^, *n* = 129	
T1	68 (52.7%)
T2	49 (38%)
T3	10 (7.8%
T4	2 (1.6%)

BMI: body mass index; OC: ovarian cancer; N/A: information not available; *: physical activity, drug and stimulant consumption, menstrual cycle phase;^#^ tumor size according to the American Cancer Society classification

### Phase 1: development of the AI algorithm

**Figure 4.**
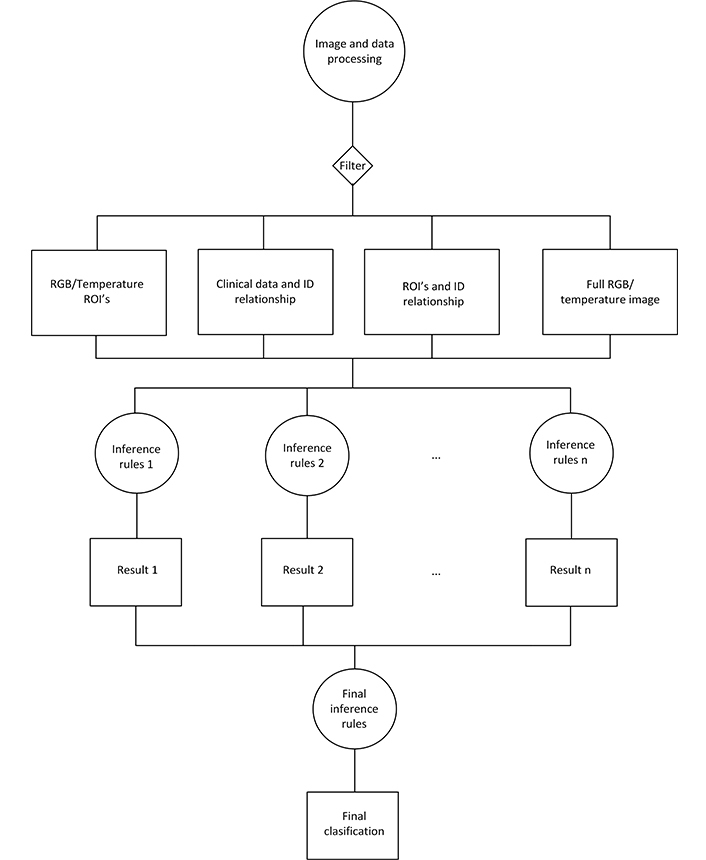
Flowchart of the implementation of the AI algorithm. RGB: red, green, blue; ROI: region of interest; ID: identity

AI algorithms developed in this phase were supplied with 2,699 patient registries, of which 2,609 were mammography BI-RADS 1,2 (patients without BC) and 90 were BI-RADS 3–5 with biopsy that confirmed BC. The 289 patient registries with BI-RADS 3–5 whose biopsy indicated benign pathology, or who were re-evaluated as BI-RADS 2 after the follow-up, were not supplied to the AI algorithms (see Figure 3). The AI algorithm with the best performance during this testing was a convolutional neural network (CNN) based on the residual net 50 (ResNet 50), with additional layers connected to an output artificial neuron (Figure 4).

The validation subset used for this algorithm was comprised of 72 patient registries, including 34 patients with BC and 38 patients without BC. The effectiveness metrics for the mammography used during phase 1 and the validation subset are shown in Table 3.

**Table 3.** Effectiveness metrics for the mammography used during phase 1 and AI validation subset

**Techniques evaluated**	**Clinical validation**		**Sensitivity**	**Specificity**	**PPV**	**NPV**	**AUC**	**F_1_ score**

	**Non-suspicious**	BC^*^						
Mammography phase 1								
BI-RADS 1,2	2,702	1	99.19%	92.03%	34.45%	99.96%	N/A	N/A
BI-RADS 3–5	234	123	π_1_					
AI validation subset								
0 (non-suspicious)	19	1	97.06%	50.0%	63.46%	95.0%	0.735	0.7674
1 (suspicious)	19	33	π_2_					

* Patients with histopathological results; N/A: not applicable

### Phase 2: clinical validation

During phase 2, additional 277 registries were generated. Together with the patient registries used with the AI algorithm validation subset, this resulted in a total of 349 that were used to validate the clinical effectiveness of the infrared-AI software. Of these, 39 corresponded to patients with confirmed BC diagnoses. The effectiveness metrics of the infrared-AI software at detecting BC cases were compared against reference mammography of the same 349 patients (Table 4).

**Table 4.** Effectiveness of the infrared-AI software and mammography in detecting BC cases

**Screening techniques**	**Clinical validation**		**Sensitivity**	**Specificity**	**PPV**	**NPV**

	**Non-suspicious**	BC^*^				
Mammography						
BI-RADS 1,2	301	0	100.0%	97.10%	81.25%	100.0%
BI-RADS 3–5^*^	9	39				
Infrared-AI software						
Th1,2 (non-suspicious)	224	2	94.87%	72.26%	30.08%	99.12%
Th3,4,5 (suspicious)	86	37				

* Patients with histopathological results

A statistical correlation was not found between the infrared-AI software test results and patient BMI (*P* = 0.697) or breast density (*P* = 0.081), nor with the presence of conditions that alters body temperature (*P* = 0.287). No other clinical variables showed a statistical correlation with the test results from the infrared-AI software (Table 5).

**Table 5.** Correlations between the infrared-AI software test results and patient characteristics

**Clinical variables**	**Value**	**Infrared-AI software result^#^ (error)**			**Infrared-AI software result^#^ (hit)**			***χ* ^2^**	***P* value**
		**FP**	**FN**	**Total**	**TP**	**TN**	**Total**		
BMI (kg/m^2^, *n* = 349)	Underweight	0	0	0	0	2	2	1.81	0.697^*^
	Normal	16	0	16	7	44	51		
	Overweight	37	1	38	15	109	124		
	Obesity	33	1	34	15	69	84		
Breast pain	No	74	0	74	17	184	201	1.97	0.16
	Yes	12	2	14	20	40	60		
Activities and conditions that affect body temperature	No	27	0	27	17	48	65	1.13	0.287
	Yes	59	2	61	20	176	196		
Breast density	A	7	0	7	5	14	19	7.67	0.081^*^
	B	65	2	67	21	168	189		
	C	12	0	12	11	42	53		
	D	2	0	2	0	0	0		
BC grade (*n* = 39)	I	-	0	-	1	-	-	0.39	0.811^*^
	II		1		19				
	III		1		13				
	N/A		0		4				
Tumor size	T1	-	0	-	19	-	-	6.02	0.120^*^
	T2		1		15				
	T3		1		2				
	T4		0		1				

# Error is the false-positive (FP) and false-negative (FN) given by the infrared-AI software; thus, hit is the true positive (TP) and true negative (TN). N/A: information not available; -: bank cell; *: *P* value calculated by Fisher’s exact test

## Discussion

In this study, an infrared-AI software developed to detect suspicious and non-suspicious cases of BC from infrared breast thermography images achieved sensitivity (94.87%) and NPV (99.12%) scores comparable to using mammography. However, the infrared-AI software’s specificity (72.26%) and PPV (30.08%) scores were inferior to mammography.

As the standard method for BC screening, mammography has been shown to reduce patient mortality rates by 25% in developed countries with well-organized screening programs [18]. However, in Mexico and across Latin America where mammography equipment and trained personnel are less available, the goal of reducing mortality through early detection of BC has not yet been achieved [19].

In this context, the infrared-AI software presented in this research study could be useful in certain countries since infrared thermography is safe, affordable, scalable, and approved by the FDA as an adjunctive tool for BC screening. Although the infrared-AI software sensitivity (94.87%) was inferior to that of the mammography used in the trials, the infrared-AI software demonstrated higher BC sensitivity compared with average mammography sensitivity in Mexico, which some studies estimate to be around 71–75% [20, 21]. It should be noted that the infrared-AI software presented in this research study was able to detect all cases of BC with T1 tumors (19 cases), thus showing its potential utility to detect early-stage malignant lesions.

Since the specificity and PPV of the infrared-AI software were lower than those of the mammography used, the use of the infrared-AI software alone is not recommended for definitive diagnosis. The low specificity of thermography has been documented in other studies [22, 23], and may be due to several benign breast pathologies producing similar thermal alterations which could result in false positives [24].

The correlation analysis showed that the infrared-AI software was not influenced by breast density, which means that the algorithm could complement mammography analyses and address the difficulty that mammography currently faces when testing young females and/or females with dense breast tissue. This is relevant in the Mexican and Latin American context, where 20% of cases of BC are detected in young women [25], reinforcing the need for more tools for BC monitoring of this age group.

### Use of AI in breast image classification

The use of AI in breast image classification has been tested in various research studies to classify mammograms [26] as well as infrared images. In 2018, Gogoi et al. [27] revised different AI algorithms for the analysis of breast infrared images, identifying a sensitivity superior to 80% in most of them, in comparison with a sensitivity of 98% in their AI method based on polynomial SVM. Zuluaga-Gomez et al. [28] developed a CNN that classified infrared breast images from a database of 57 patients; by using “data augmentation” and “hyperparameters tuning”, their AI algorithm obtained a sensitivity of 92% and an accuracy of 94%.

It is important to note that Zuluaga-Gomez et al. [28] and other works used publicly available databases for their research, mainly the Database for Mastology Research (DMR) database (287 patients) [29], and the Department of Biotechnology, Tripura University and Jadavpur University (DBT-TU-JU) database (100 patients) [30]. The present research study was made in a specialized medical center for breast pathologies (FUCAM A.C.) which allowed the research team to build a large proprietary database of infrared images (20,022 images from 3,337 patients) with respective clinical, radiological, and histopathological patient information. Under the scope of some recent review publication works [31, 32], this research is one of the largest combining AI and thermography.

Additionally, unlike many studies, this research was not limited to the evaluation of an AI algorithm but also carried out clinical validation of the software itself. In a similar clinical trial, Rassiwala et al. [33] performed infrared, clinical exploration, radiologic and histopathologic tests with 1,008 female patients. Their methodology for analyzing thermal images was based on the temperature gradient calculation, and those with test results of delta T (ΔT) > 2.5 were classified as abnormal. They reported a sensitivity of 97.6%, specificity of 99.17%, PPV of 83.67%, and NPV of 99.89%.

### Research limitations

The low capacity of the infrared-AI software to differentiate between benign and malignant pathologies, with a specificity of 72%, could be due to the exclusion of 289 patients with benign pathology from the AI algorithm’s development. This decision was made due to the research’s principal goal, which was to increase the algorithm’s BC sensitivity. Future studies could investigate whether introducing benign pathology to the AI algorithm development increases specificity without reducing sensitivity. In addition, the CNN architecture used in the infrared-AI software increases its efficiency when more data is provided [34], therefore continuing to collect data could improve the clinical effectiveness of the infrared-AI software.

The authors acknowledge a few methodological limitations of this research. Firstly, the improbable 100% sensitivity and NPV for the used mammography are likely due to an inability to measure false negatives from FUCAM A.C.’s mammography. Similarly, the research’s scope did not include a third diagnosis test for instances where the infrared-AI software detected a suspicious case, but the mammography did not. A third diagnosis test was not implemented here because it was considered unethical to perform a biopsy on patients when the standard BC screening technique considered the patient not to be at risk of malignancy.

### Future work

In the future, the infrared-AI software could be evaluated with a larger sample of patient registries and following a trial design that removes the methodological limitations described above. In addition, future work could evaluate the efficiency and cost-effectiveness of a BC screening strategy that integrates the infrared-AI software with mammography and ultrasound for females aged 40 years and above, and one that integrates the infrared-AI software with ultrasound for younger females.

### Conclusions

In this work, an infrared-AI software developed to detect BC from infrared breast thermography images (infrared-AI software) showed high sensitivity (94.87%) and high NPV (99.12%), but lower specificity (72.26%) and PPV (30.08%) compared to mammography, the standard BC screening technique. The infrared-AI software is therefore proposed as a complementary tool for BC screening, but not for definitive diagnosis. Increasing the amount and range of patient data that the AI algorithm uses to build its predictive model, as well as the amount of patient data available for clinical validation, will improve its efficiency, accuracy, and potential value as a complementary BC screening tool.

## Abbreviations

AI: artificial intelligence
BC: breast cancer
BI-RADS: breast imaging reporting and data system
BMI: body mass index
CNN: convolutional neural network
FUCAM A.C.: Breast Cancer Foundation
NPV: negative predictive value
PPV: positive predictive value

## Supplementary material

The supplementary material for this article is available at: https://www.explorationpub.com/uploads/Article/file/1002135_sup_1.pdf.

## Declarations

### Availability of data and materials

The electronic patient record data used to support the findings of this research are restricted by FUCAM A.C. in order to protect patient’s privacy, also, the database of infrared images generated in this trial are property of Heathcore SAPI de CV. Requests for accessing the datasets should be directed to [Eva Ruvalcaba-Limon, evaruvalcaba@yahoo.com.mx] and [Pedro A. Sánchez-Méndez, pedro.sanchez@thermy.com.mx] if the researcher meets the criteria for access to confidential data.

### Copyright

© The Author(s) 2023.
