# Supplementary material for: Development and validation of an infrared-artificial intelligence software for breast cancer detection
Source: Explor Target Antitumor Ther. 2023 Apr 27;4(2):294–306. doi: 10.37349/etat.2023.00135 (PMC10189354; doi:10.37349/etat.2023.00135)

Appendix A: questionnaire about clinical data and prior factors to the infrared test

Registry of clinical data and prior factors to the infrared test

File: ________________________Date: ________/________/____________

Age: ___________ years old Weight: _____________kg

Height: ______________m

Waist circumference: _________ cm Hips circumference: _________cm

Bra cup: ________

Last menstruation age: _________years old Current phase of the menstrual cycle: ________________

Have you ever:	**Yes**	**No**
Had a family history of breast or ovary cancer?	○	○
From which relative(s)? ____________________________		
Had received radiologic treatment on the upper body segment?	○	○
In the last 24 hours, have you:		
Altered your sleep or eating habits?	○	○
Felt any physical discomfort in your breasts*?	○	○
Taken any kind of medicament or drug?	○	○
Which one (ones)? _______________________		
In the last 6 hours, have you:		
Performed vigorous physical activity?	○	○

Applied crems, gels, spray, etc., in the breast region? ○ ○

Consumed coffee, alcohol, tobacco, or any other stimulant? ○ ○

Which one(ones)? ________________________________

*Point out the area where you felt physical discomfort:

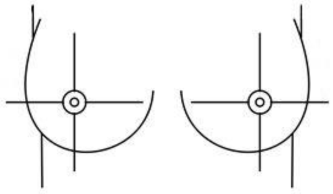

Supplement: Supplementary file 1 [file etat-04-1002135_sup_1.pdf]
